# Two clones, two paths: divergent intrinsic xylem traits and hydraulic responses to prolonged drought in poplar

**DOI:** 10.1093/aob/mcag254

**Published:** 2026-08-12

**Authors:** Niccolò Tricerri, Rachele Gamba, Cristina Morabito, Silvia Cavalletto, Francesco Maimone, Pier Mario Chiarabaglio, Andrea Carra, Alan Crivellaro, Francesca Secchi

**Affiliations:** Department of Agriculture, Forest and Food Sciences, University of Turin, Largo Paolo Braccini 2, Grugliasco 10095, Italy; University School for Advanced Studies IUSS Pavia, Piazza della Vittoria 15, Pavia 27100, Italy; Department of Agriculture, Forest and Food Sciences, University of Turin, Largo Paolo Braccini 2, Grugliasco 10095, Italy; Department of Agriculture, Forest and Food Sciences, University of Turin, Largo Paolo Braccini 2, Grugliasco 10095, Italy; Department of Agriculture, Forest and Food Sciences, University of Turin, Largo Paolo Braccini 2, Grugliasco 10095, Italy; Department of Agriculture, Forest and Food Sciences, University of Turin, Largo Paolo Braccini 2, Grugliasco 10095, Italy; Council for Agricultural Research and Economics, Research Centre for Forestry and Wood, Strada Frassineto 35, Casale Monferrato 15033, Italy; Council for Agricultural Research and Economics, Research Centre for Forestry and Wood, Strada Frassineto 35, Casale Monferrato 15033, Italy; Department of Agriculture, Forest and Food Sciences, University of Turin, Largo Paolo Braccini 2, Grugliasco 10095, Italy; Forest Biometrics Laboratory, Faculty of Forestry, ‘Stefan cel Mare’ University of Suceava, Str. Universitatii 13, Suceava 720229, Romania; Department of Agriculture, Forest and Food Sciences, University of Turin, Largo Paolo Braccini 2, Grugliasco 10095, Italy

**Keywords:** *Populus × canadensis*, clone, prolonged drought, embolism, wood anatomical trait

## Abstract

**Background and Aims:**

Climate change is increasing the frequency and intensity of droughts, challenging the growth, hydraulic function and survival of woody plants. Poplar is a fast-growing species widely cultivated for biomass and cellulose production, but it is also highly sensitive to water deficit, with substantial variation among species and clones.

**Methods:**

This study compared the drought responses of two *Populus × canadensis* clones, ‘Tucano’ and ‘San Martino’, under gradually imposed, prolonged water stress. Physiological traits and intrinsic wood anatomical characteristics revealed different strategies between clones for coping with drought.

**Key Results:**

Compared with the ‘San Martino’ clone, the ‘Tucano’ clone exhibited faster stomatal closure under moderate stress and a greater resistance to embolism formation. The resistance was further supported by specific intrinsic wood anatomical traits, including higher vessel density, increased vessel clustering, greater theoretical hydraulic conductivity, thicker cell walls and higher wood density. Conversely, the delayed stomatal closure, the less negative P50 value and the distinct xylem anatomical structure observed in ‘San Martino’ suggest a significantly higher vulnerability to prolonged water deficit.

**Conclusions:**

‘Tucano’ adopts an effective drought-avoidance strategy, integrating stomatal regulation, intrinsic wood anatomy and carbon allocation to balance hydraulic safety with growth and wood quality. The study highlights specific traits as key determinants of drought resistance and supports the selection of a specific clone for poplar cultivation in future climate scenarios characterized by water scarcity.

## INTRODUCTION

Global climate change is increasing the frequency, intensity and duration of drought, making it one of the dominant abiotic stressors for woody plants (Rosso *et al.*, 2023). Rising maximum temperatures and altered precipitation patterns further exacerbate drought stress worldwide and challenge tree survival (McCarthy, 2001; Trivedi *et al.*, 2025). These climatic changes intensify the physiological stress on trees during drought, because increasing potential evapotranspiration is constrained by limited soil water availability (Allen *et al.*, 2010; Adams *et al.*, 2017).

Trees respond to drought through a wide range of morphological, physiological and anatomical strategies (Attia *et al.*, 2015; Rosso *et al.*, 2023). For example, stomatal closure limits water loss, which simultaneously reduces CO_2_ uptake and assimilation, thereby affecting non-structural carbohydrate balances (Nawaz *et al.*, 2025) through the depletion of reserves and reduced growth. In addition, intrinsic xylem anatomical traits, such as conduit diameter, their connectivity, density and pit properties, can further enhance drought resistance (Cai and Tyree, 2010; Losso *et al.*, 2017).

As soil water potential declines and transpiration demand increases, stem water potential can drop sufficiently to allow entry of air through pits into neighbouring conduits, which will no longer be able to transport water if embolism occurs (Fahad *et al.*, 2017). If this hydraulic damage is not repaired, tissue damage, tree desiccation and reduced productivity and vitality may occur (Nadal-Sala *et al.*, 2024). Therefore, the resistance of the tree to xylem embolism is crucial for preventing hydraulic failure. Nevertheless, considerable variation in embolism formation exists among tree species, largely reflecting differences in ecological adaptation and environmental conditions (Fichot *et al.*, 2015). Xylem embolism resistance is commonly represented using the hydraulic P50 value. P50 is taken as representative of the ability of a species to resist embolism, because it indicates the stem water potential at which 50 % of hydraulic conductance is lost. When water potential drops below P50, water transport becomes severely impaired. Xylem anatomical traits associated with embolism resistance, such as thick pit membranes, reinforced conduit walls and increased fibre-wall area, enhance hydraulic safety (Cai and Tyree, 2010; Losso *et al.*, 2017) but require substantial carbon investment, highlighting a trade-off between xylem resistance and carbon allocation.

The metabolic cost of hydraulic safety is compounded further by the essential role of non-structural carbohydrates (NSCs), because they play a central role in maintaining plant hydraulic function during drought. Until water deficiency becomes too limiting, NSCs support xylogenesis by enabling the formation of new xylem (and phloem) and sustaining hydraulic capacity despite embolism or mechanical damage in older vessels (Tomasella *et al.*, 2019). In addition, soluble carbohydrates sustain metabolism when photosynthesis is limited and contribute to the restoration of hydraulic conductivity through osmotically driven refilling of embolized vessels (Secchi and Zwieniecki, 2011; Pagliarani *et al.*, 2019).

Poplar is one of the most productive species for the woody biomass and cellulose supply chain, with plantations covering ∼31.4 million ha worldwide (Corona *et al.*, 2018). China and Europe (particularly France, Italy and Spain) are the main producers, with the Po Valley in northern Italy representing one of the most important regions for the quality of production, where poplar plantations cover 434,000 ha (Marchi *et al.*, 2022). Approximately 70 % of the poplar nursery stock in Italy comes from the Piedmont region (Rizza *et al.*, 2025), where genetic improvement programmes have led to the development of new poplar clones characterized by strong rooting capacity, rapid growth rates, disease tolerance, vigorous resprouting ability and high-quality wood production (Corona *et al.*, 2018). Although these clones exhibit tolerance to major biotic adversities, their responses to abiotic stresses are not yet fully characterized.

Different poplar genotypes exhibit variability in both anatomical traits and physiological responses during drought (Rosso *et al.*, 2023). For instance, *Populus × canadensis* Moench and *Populus cathayana* Rehder differ in carbohydrate production and accumulation and in intrinsic water-use efficiency (iWUE) (Cao *et al.*, 2014). Variability is also observed, following drought imposition, among clones of the same species (*Populus nigra* L.), which differ in biomass, biometric traits, behaviour of stomata, iWUE and sugar allocation (Regier *et al.*, 2009). Moreover, male *P. cathayana* trees exhibit specific anatomical traits, including a higher number of mid-sized vessels with thicker cell walls and greater vessel grouping, suggesting greater drought resistance than female trees (Liu *et al.*, 2022).

Poplar genotypes differ widely in how they balance the trade-off between drought resilience and growth. A study of 33 poplar genotypes revealed that those that achieved the highest biomass yield in optimal water conditions tended to exhibit the largest reductions in biomass productivity when exposed to drought, although these patterns can occur both within and among poplar species (Monclus *et al.*, 2006).

Although poplar is widely recognized as one of the most drought-sensitive tree species, large variation in drought tolerance has been observed among species, genotypes and clones (Monclus *et al.*, 2006; Silim *et al.*, 2009). Therefore, identifying whether a given clone is better adapted to drought is particularly relevant in areas where poplar cultivation plays a key role in the local economy and is expected to face increasingly frequent and prolonged drought events in the near future (Rosso *et al.*, 2023). In this study, we aimed to determine whether two *P. × canadensis* Moench clones, ‘Tucano’ and ‘San Martino’, exposed to a similar natural timing of water stress occurrence exhibit different drought response strategies. Therefore, the physiological responses of the two clones were characterized during a prolonged drought, whereas xylem anatomical traits were analysed in control plants to assess intrinsic structural differences that might explain the contrasting responses of the clones to water deficit. Specifically, we addressed the following questions.

1. Do the wood anatomical, physiological and biochemical responses of ‘Tucano’ and ‘San Martino’ differ in drought conditions?
2. Which physiological mechanisms and anatomical traits confer greater drought tolerance to one clone compared with the other?

In this context, our study revealed that the ‘Tucano’ clone exhibited a distinct wood anatomical structure that was directly correlated with superior physiological tolerance to drought. Our results deepen our understanding of poplar responses to prolonged drought under climate change and provide new insights that might support more effective management and the selection of more drought-resilient clones for future plantation systems.

## MATERIALS AND METHODS

### Plant material and experimental design

Cuttings of *P. × canadensis* (*P. deltoides × P. nigra*) clones ‘San Martino’ and ‘Tucano’, characterized by high tolerance to major biotic adversities, were released by the CREA-Forestry and Wood (CREA-FL, Casale Monferrato, NW Italy). Plant material for this study was provided by CREA-FL. Cuttings (20 cm long) were soaked in water for 7 days, then planted in 10 L pots filled with a peat-based substrate (Turco Silvestro, TS3). Plants were grown for 2 months in a greenhouse in semi-controlled conditions before the experiment.

A total of 94 2-month-old poplars were irrigated daily to pot capacity until the onset of drought treatments. At day 0, mean stem diameter and height were 8.7 ± 1.2 mm and 80.9 ± 7.4 cm for the ‘San Martino’ clone and 7.2 ± 1.1 mm and 93.1 ± 12.4 cm for the ‘Tucano’ clone, respectively. Plants of both clones were divided into a control group and a drought-stressed group. Sixteen plants (eight per clone) were maintained in well-watered conditions (CTR), while the remaining 78 plants (45 ‘Tucano’ and 33 ‘San Martino’) were subjected to a progressive drought starting on day 0 (Fig. 1). Drought was imposed by restoring 40 % of the water lost on the previous day, with pot weight recorded every morning. Pot capacity was determined after drainage. Specifically, plants were irrigated, and excess water was allowed to drain freely from the pots until gravitational water had been lost. Pot weight at this stage was considered as pot capacity and was subsequently used as the reference value for irrigation treatments. The drought treatment continued until the stem water potential reached approximately −1.5/−2 MPa. Greenhouse daily temperature, light intensity and air relative humidity during the growing and experimental periods are reported in Supplementary Data Fig. S1.

**Fig. 1.**
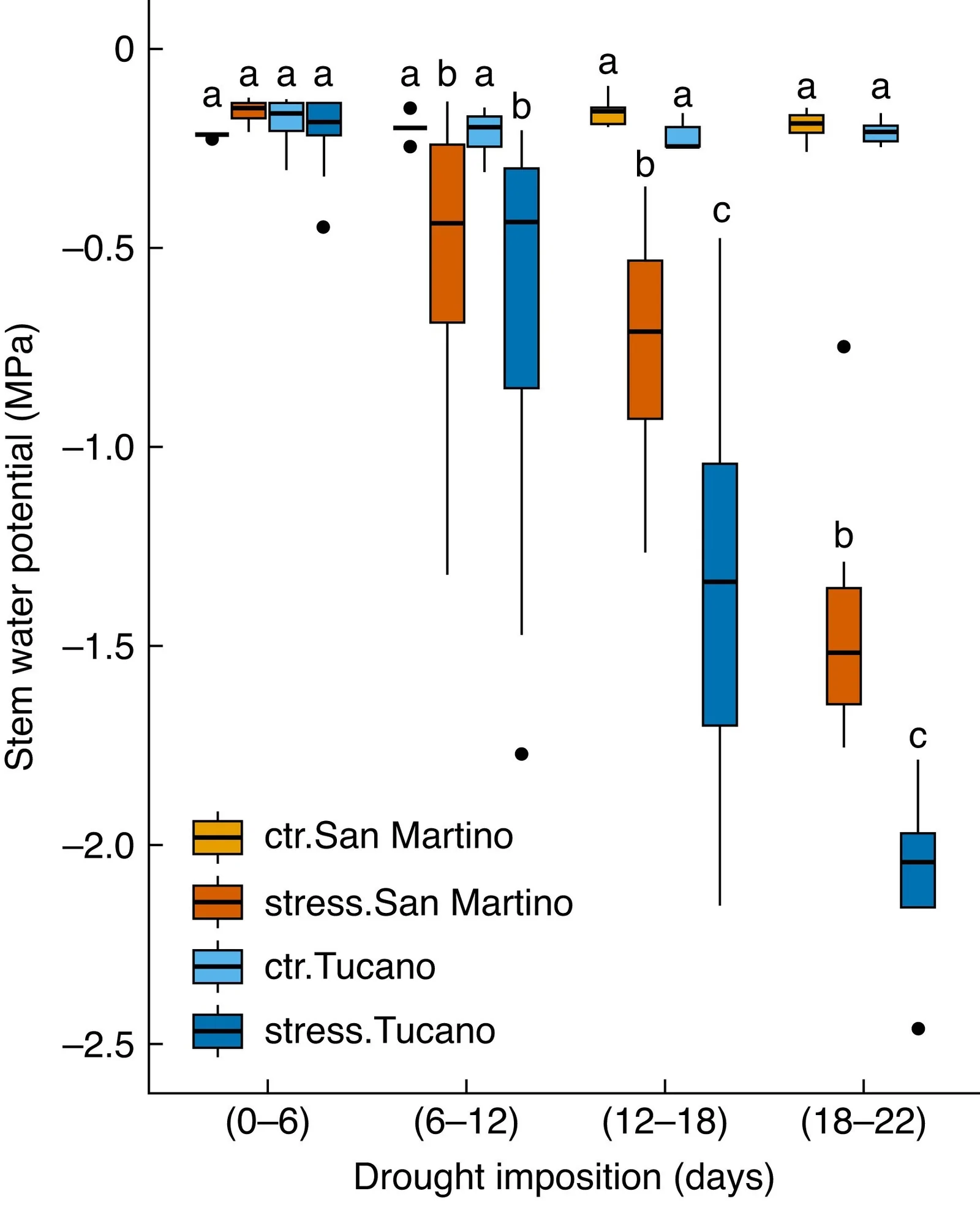
Temporal dynamics of stem water potential (Ψ_stem_) in ‘Tucano’ and ‘San Martino’ clones subjected to progressive drought. One-way ANOVA suggests significant differences during the imposition of the stresses (*P* < 0.05). Letters denote homogeneous groups based on the Waller–Duncan *post hoc* test for each bin (*n* = 6 days). The statistical results are reported in Supplementary Data Table S1.

The stem diameter of 23 stressed plants (15 ‘Tucano’ and 8 ‘San Martino’) and 16 control plants (8 per clone) was recorded at regular intervals throughout the experiment (Supplementary Data Fig. S2). A total of 55 plants (30 ‘Tucano’ and 25 ‘San Martino’) were sampled destructively at different stem water potentials to construct vulnerability curves and assess carbon allocation. Leaf gas exchanges were measured in the morning on five plants per treatment for each clone. Control plants were monitored throughout the experimental period and sampled at the end of the experiment (total growth period of 88 days) for wood anatomical analyses (ten plants in total, five per clone).

### Physiological measurements

Stem water potential was measured on the same poplar plants used for the percentage loss of conductivity (PLC) assessments. For each tree, a fully expanded leaf was enclosed in a humidified plastic bag wrapped in aluminum foil for ≥20 min to stop transpiration. After leaf excision, samples were allowed to equilibrate for ∼20 min, and stem water potential (ψ_stem_) was then determined using a Scholander-type pressure chamber (model 1505D, PMS Instruments, OR, USA).

Leaf gas exchanges were measured on fully expanded leaves using a portable infrared gas analyser (LCpro-SD system, ADC BioScientific Ltd, Hoddesdon, UK) equipped with a 6.25 cm^2^ chamber. During measurements, the CO_2_ concentration was checked to be stable between 420 and 450 ppm, and chamber temperature was set to 25 °C. All measurements were conducted in the morning (08:00–10:00 h) under natural light in the greenhouse. The parameters were monitored daily on randomly selected plants (*n* = 3–4) of each group and clone throughout the experimental period. iWUE was calculated as the ratio of net CO_2_ assimilation (*A*; in micromoles of carbon dioxide per square metre of leaf area per second) to stomatal conductance (*g*_s_; in moles of H_2_O per square meter of leaf surface per second).

Embolism formation was quantified indirectly through measurements of stem hydraulic conductivity before and after flushing stem segments at moderate to high pressure in order to remove any air bubbles potentially present within the conduits, following the approach described by Secchi and Zwieniecki (2010). Briefly, poplar stems of the two clones were cut under water to prevent embolism formation owing to air entry into xylem vessels. From each stem, three segments ∼4 cm in length were excised 20–30 cm above the initial cut. The vessel length of three species of the genus *Populus* has been estimated by Cai *et al.* (2010) for different vessel diameter size. They found that large-diameter vessels are longer than small-diameter vessels. The largest vessel diameter measured was 60 cm, and the average vessel length, determined using the silicone rubber injection method, was ∼4–5 cm. Initial hydraulic conductance (*K*_i_) was determined gravimetrically by measuring the flow rate of a filtered 10 mM KCl solution supplied from a reservoir placed on an analytical balance (CPA 225D, Sartorius; ±0.01 mg) and connected to the stem segment via a plastic tube. Samples were submerged in a water bath ∼10 cm below the water level of the reservoir. Once a steady flow was reached (within a few minutes), embolisms were removed by flushing the segments with water at ∼2 bar for 20 s, after which maximum hydraulic conductance (*K*_max_) was measured.

The percentage loss of conductivity (PLC) was calculated as:


(1) $$\mathrm{PLC}=100\times \left(K_{\max}-K_{i}\right)/K_{\max}$$

The relationship between ψ_stem_ and PLC was fitted with a four-parameter logistic curve (Secchi and Zwieniecki, 2014) and used to determine the P50 values. Xylem vulnerability curves were constructed for both clones, and the corresponding P50 parameters of the fitted curves were compared using the corrected statistical *Z*-test for the equality of regression coefficients (Paternoster *et al.*, 1998).

### Wood anatomical measurements

Wood anatomical analyses were conducted on 3-month-old control plants, randomly selected from each clone (*n* = 5 per clone). A 5-cm-long stem segment was cut from each selected control plant at the end of the experiment, preserved in a fixative solution (Formalin Free Tissue Fixative, Sigma-Aldrich) and stored at room temperature until sectioning. To account for axial variation in wood anatomical traits (Anfodillo *et al.*, 2013; Olson *et al.*, 2021), a 5-cm-long stem segment was consistently sampled at a fixed distance of 40 cm below the main stem apical bud. Transverse stem anatomical sections (∼20 µm thick) were obtained using a sliding microtome (Reichert-Jung, Optische Werke AG, Wien, Austria). Sections were double-stained for 3–5 min with a 1:1 mixture of freshly prepared Safranin (1 % in distillated water) and Astra Blue (0.5 % in distillated water with 0.2 % acetic acid) (Gärtner and Schweingruber, 2013). This double-staining procedure stains lignified cell walls red and less lignified cell walls blue. After staining, sections were rinsed with water and dehydrated through an ethanol concentration gradient (from 50 to 75 to 95 % v/v), then embedded in glycerol under a coverglass. Images of sections were acquired at ×100 magnification using a digital camera (CANON EOS700D, Bovenkerkerweg 59, 1185 XB, Amstelveen, The Netherlands) mounted on an optical microscope (Olympus BX 51, Tokyo, Japan), and the selected region of interest of the xylem area was then analysed using CARROT (Resente *et al.*, 2021, 2024) and ImageJ software (FIJI distribution package, IMAGEJ2 v.2.16.0/1.54p; Schindelin *et al.*, 2012) (see Supplementary Data Materials; Supplementary Data Fig. S3). The anatomical measurements obtained from the analysed regions were subsequently scaled to the total xylem cross-sectional area of each entire cross-section, allowing comparisons among individuals on a whole-cross-section basis. The following wood anatomical traits were analysed: (1) vessel lumen area (LA; in micrometres squared); (2) vessel lumen diameter (*d*; in micrometres); (3) vessel density (V_density_; number per millimetre squared); (4) vessel multiple fraction (Fvm; ratio of grouped vessels to total number of vessels); (5) percentage of vessels in two-vessel groups (2-vessel groups; as a percentage); (6) percentage of vessels in three-vessel groups (3-vessel groups; as a percentage); (7) percentage of vessels in four-vessel groups (4-vessel groups; as a percentage); and (8) cell wall thickness (vessels and fibres; in micrometres) measured along the radial direction as the double wall of two adjacent cells.

Mean hydraulic diameter (*D*_h_; in micrometres) was calculated according to eqn (2):


(2) $$D_{h}=(⟮\sum_{i=1}^{n}d^{4})/n{)}^{0.25}$$

where *d* is the diameter of the n vessel, and *n* is the number of vessels measured in the sample (Tyree and Zimmermann, 2002). The theoretical hydraulic conductivity (Kh_theo_; in metres cubed per megapascal per second) was estimated according to the Hagen–Poiseuille equation [eqn (3)]:


(3) $$Kh_{\mathrm{theo}}=\left(\pi \times D_{h}^{4}\right)/128\eta$$

where *D*_h_ is specific for each plant, and *η* is the dynamic viscosity of water at 25 °C at atmospheric pressure (8.9 × 10^−10^ MPa s) (Tyree and Zimmermann, 2002). Then, the specific theoretical hydraulic conductivity (Ks_theo_) was calculated by dividing theoretical hydraulic conductivity by the xylem area.

The packing limit represents the maximum number of vessels that can be placed in an area for a given mean vessel cross-sectional area (McCulloh *et al.*, 2010). The geometric packing limit was calculated assuming square packing, as the maximum number of conduits of a given diameter per millimetre squared, according to: packing limit = 1/*d*^2^.

For each control plant of both clones, the diameter corresponding to the average lumen cross-sectional area was calculated as the area-weighted mean conduit diameter (dA), as in eqn (4) (McCulloh *et al.*, 2010; Crivellaro *et al.*, 2012):


(4) $$\mathrm{dA}=(\Sigma d^{2}/n{)}^{\frac{1}{2}}$$

Wood density was determined on debarked stem segments of the same control plants used for other anatomical analyses. Before determining fresh volume, samples were submerged in distilled water for 24 h to maximize water content. Fresh wood volume was then measured using a revised Archimedes displacement method (Hughes, 2005). Each segment was immersed in a beaker of distilled water placed on a balance, and the mass of displaced water was recorded. Room temperature was measured during the process, and volume was calculated using the corresponding water density from standard tables. Samples were subsequently dried at 60 °C for 48 h and weighed to obtain dry mass. Wood density was calculated as the ratio of dry mass to fresh volume (in grams per centimetre cubed).

### Quantification of soluble sugar and starch

Stem tissues were collected from the same plants that were used to determine PLC. Samples were oven-dried at 60 °C for 48 h, then ground to fine powder (particle size of <0.15 mm) using a tissue lyser system (TissueLyser II, Qiagen). Ground tissues were stored at room temperature until analysis of starch and soluble sugars. Soluble NSCs were quantified following the protocol described by Quentin *et al.* (2015) and Landhäusser *et al.* (2018) and modified by Gargiulo *et al.* (2024). Briefly, dried samples (15 ± 1 mg) were suspended in 80 % (v/v) ethanol and incubated at 80 °C for 30 min. After centrifugation at 16.120 xg for 5 min, the supernatant was separated from the pellet. The extraction step was repeated twice. Samples containing soluble NSCs were placed in an oven at 55 °C overnight. Once the evaporation of ethanol was completed, the resulting crystallized carbohydrates were suspended in distilled water. Soluble NSC content was determined using the anthrone assay (Leyva *et al.*, 2008). Absorbance was measured at 620 nm with a 96-well microplate reader spectrophotometer (iMark Microplate Absorbance Reader, BioRad) and compared against a glucose standard curve. Results were expressed as milligrams per gram dry weight of glucose.

Starch concentration was determined using an enzymatic assay kit (STA20, Sigma Aldrich), following the modifications described by Morabito *et al.* (2025). Absorbance was read at 540 nm, and starch concentration was calculated based on a glucose standard curve and expressed as milligrams of glucose equivalents per gram of dry weight.

### Statistical analysis

All the analyses were performed using R (R Core Team, 2024) in the RStudio Integrated Development Environment (RStudio Team, 2023). With regard to the weight of the pots, the data were analysed statistically using the Kruskal–Wallis test followed by Dunn’s multiple-comparison *post hoc* test (Kruskal and Wallis, 1952). For gas exchange measurements, treatment effects were evaluated using one-way ANOVA followed by Waller–Duncan multiple comparison tests for each date (Waller and Duncan, 1969). Differences in wood anatomical parameters between the two clones were assessed using a two-tailed *t*-test (Student, 1908).

## RESULTS

### Physiological measurements

Both poplar clones were subjected to a prolonged drought stress. ‘Tucano’ plants reached a stem water potential (Ψ_stem_) of −2.0 ± 0.3 MPa within 22 days, whereas ‘San Martino’ poplars achieved a value of −1.5 ± 0.3 MPa over the same period. When the ‘San Martino’ leaves reached this stem water potential value, they started to fall. This leaf phenomenon was not observed in the ‘Tucano’ clone.

Differences in Ψ_stem_ between the drought-stressed clones were already evident after 12 days of stress, with ‘Tucano’ exhibiting a faster decline (drought imposition bin 12–18 days: *F* = 22.02, d.f. = 3, *P* = 9.86 × 10^−8^) and maintaining lower Ψ_stem_ values until the end of the experiment. Both control groups maintained constant Ψ_stem_ values of −0.12 ± 0.05 MPa over time, and no statistical differences were observed between well-watered clones (Fig. 1).

Although the two stressed groups had similar pot weights at the beginning of the experiment (Fig. 2A), ‘Tucano’ plants subsequently lost water at significantly higher rates than the ‘San Martino’ group (1 day after the onset of drought treatment: *F* = 52.710, d.f. = 3, *P* = 2.11 × 10^−11^). This was particularly clear during the first 6 days of treatment, when evapotranspiration levels were significantly higher (see statistical results in Supplementary Data Table S1). Consequently, ‘Tucano’ plants reached the lower end of soil water content earlier, reducing total evapotranspiration, whereas ‘San Martino’ poplars were still losing water on day 13 (13 days after the onset of drought treatment: *t* = 5.690, d.f. = 51.9771, *P* = 5.90 × 10^−7^; Fig. 2B) Specifically, water loss during the first 6 days was approximately ∼263 and ∼193 g day^−1^ for ‘Tucano’ and ‘San Martino’ plants, respectively, and between 6th and 12th days it was ∼100 and ∼76 g day^−1^, respectively. However, starting on the 12th day, ‘San Martino’ exhibited higher evapotranspiration than ‘Tucano’ (Fig. 2B). By the end of drought treatment, however, both poplar clones exhibited similar total water loss (∼70 %; Fig. 2A; Supplementary Data Table S1).

**Fig. 2.**
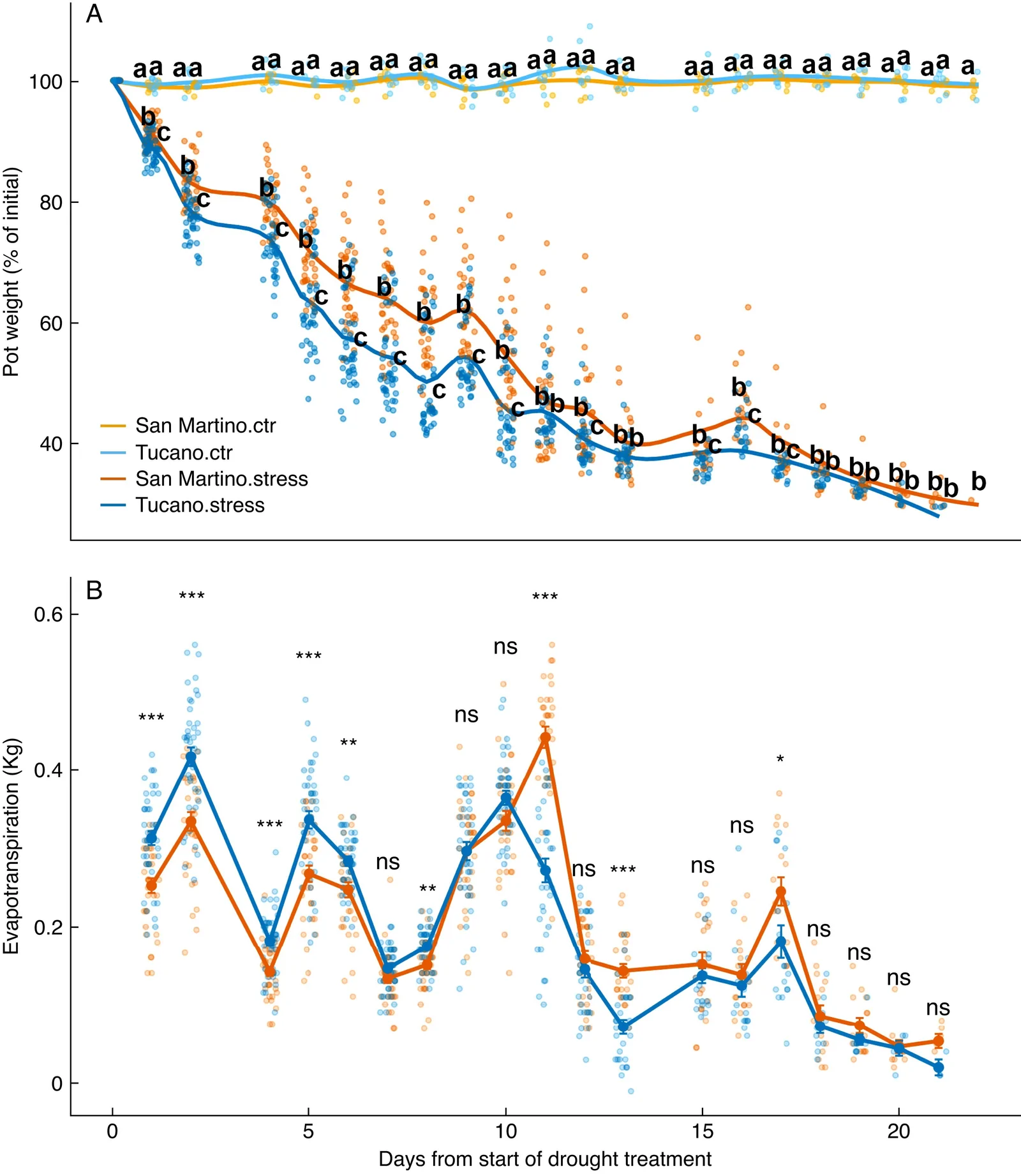
Temporal dynamics of relative pot weight (as a percentage; A) and daily evapotranspiration rates (B) for ‘Tucano’ and ‘San Martino’ clones during progressive drought. Different lowercase letters denote significant differences between time treatments within days based on the Kruskal–Wallis test followed by Dunn’s *post hoc* test. Asterisks indicate significant differences between clones at specific time points according to a paired *t*-test (*P < 0.05, **P < 0.01 and ***P < 0.001; ns, not significant). The statistical results are reported in Supplementary Data Table S1.

At the beginning of the experiment, the two clones had similar biometric parameters (see the Materials and Methods section and Supplementary Data Fig. S2). Over time, ‘Tucano’ grew more than ‘San Martino’ in both well-watered and drought conditions (Supplementary Data Fig. S2; Supplementary Data Table S2). At the end of the experiment, in well-watered conditions, stem diameter increased by 18 and 14 % for ‘Tucano’ and ‘San Martino’, respectively. Drought reduced growth in both clones, with a stronger effect observed in ‘San Martino’ (27 days after the onset of the experiment: *F* = 3.511 × 10^+31^, d.f. = 3, *P* = <2 × 10^−16^). The increase in stem diameter of drought-treated plants was limited only during the first 12 days of the experiment, when the stem water potential had not yet reached highly negative values. At the end of stress experiment, ‘Tucano’ showed an increment of ∼9.7 %, whereas ‘San Martino’ increased stem diameter by only 3 % (Supplementary Data Fig. S3).

Net photosynthesis (*A*) and stomatal conductance (*g*_s_) exhibited similar temporal responses to progressive stress in both clones, declining as stress progressed until stomatal closure occurred and carbon assimilation was almost completely suppressed (statistical results for time factor for *A*: *F* = 88.993, d.f. = 3, *P* = <2 × 10^−16^; statistical results for time factor for *g*_s_: *F* = 26.021, d.f. = 3, *P* = 1.94 × 10^−13^). ‘Tucano’ and ‘San Martino’ showed complete stomatal closure at the end of the experiment; however, ‘Tucano’ exhibited a substantial reduction in *g*_s_ and *A* after 6–12 days of drought (*A*: *F* = 11.37, d.f. = 3, *P* = 7.22 × 10^−6^; *g*_s_: *F* = 13.59, d.f. = 3, *P* = 1.08 × 10^−6^), whereas ‘San Martino’ reached similar values after 12–18 days of stress (Fig. 3A, B). Well-irrigated poplars of both clones showed similar *A* and *g*_s_ values over time.

**Fig. 3.**
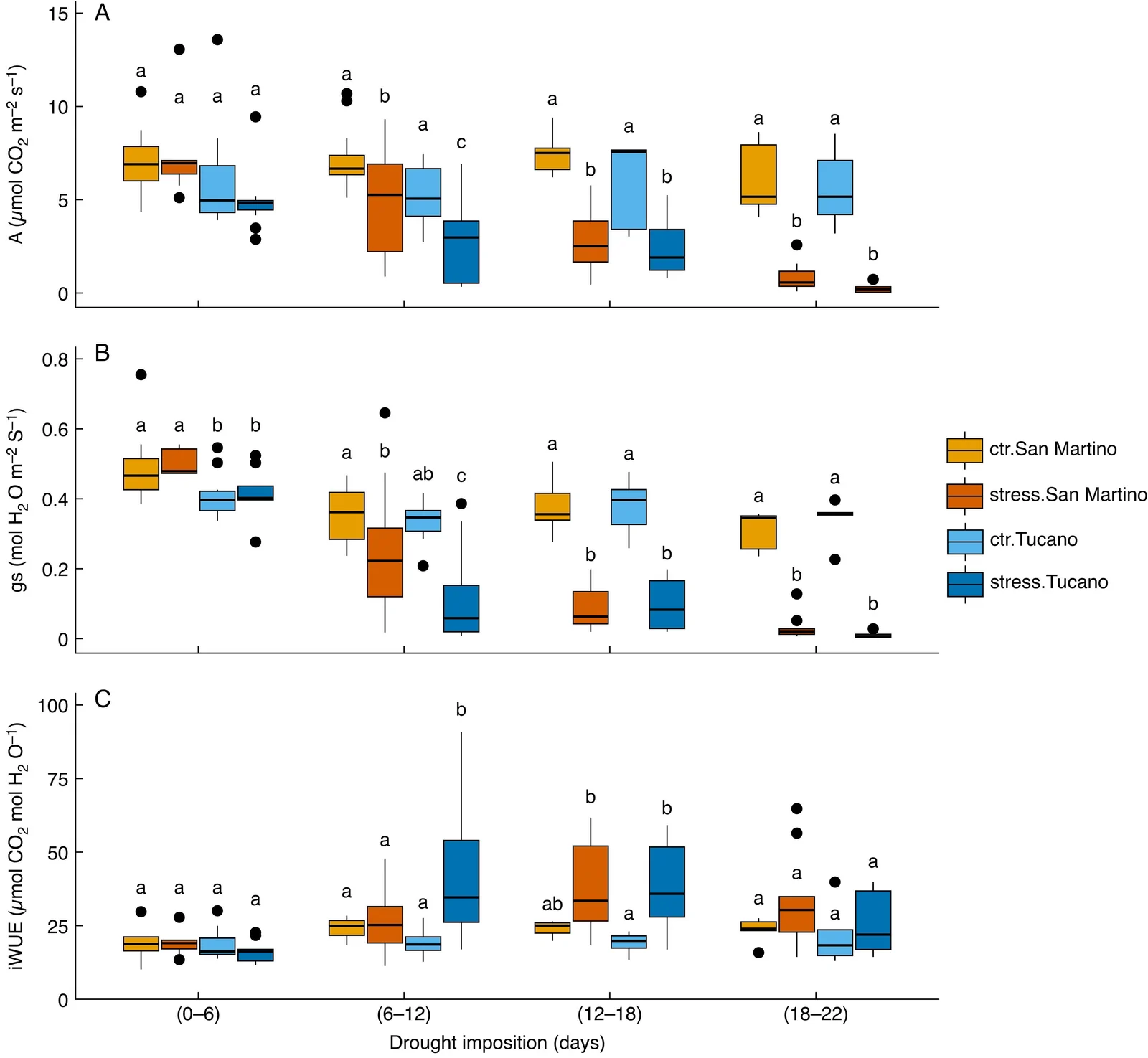
Temporal dynamics of leaf gas exchanges in ‘Tucano’ and ‘San Martino’ clones well watered and subjected to progressive drought. (A) Net photosynthesis (*A*). (B) Stomatal conductance (*g*_s_). (C) Intrinsic water-use efficiency (iWUE). One-way ANOVA suggests significant differences during the imposition of the stresses (*P* < 0.05). Letters denote homogeneous groups based on the Waller–Duncan *post hoc* test for each bin (*n* = 6 days). The statistical results are reported in Supplementary Data Table S1.

Drought-stressed plants exhibited more pronounced fluctuations in iWUE over time (statistical results for time factor for iWUE parameter: *F* = 10.649, d.f. = 3, *P* = 2.38 × 10^−6^). Furthermore, ‘Tucano’ plants displayed a significantly higher iWUE than ‘San Martino’ (1.65-fold more) starting after 6–12 days of stress (iWUE: *F* = 8.501, d.f. = 3, *P* = 0.000105), indicating a greater capacity to regulate water loss while maintaining carbon assimilation. Between the 13th and 18th days, the stressed ‘Tucano’ plants maintained a high iWUE (iWUE: *F* = 3.935, d.f. = 3, *P* = 0.0193), while the iWUE of the stressed ‘San Martino’ plants increased but was not significantly different from that of the ‘San Martino’ control plants (Fig. 3C).

Both well-watered clones showed similar levels of native embolism, averaging ∼13 and 16 % for ‘Tucano’ and ‘San Martino’, respectively (Fig. 4). Increasing water stress resulted in additional losses of stem hydraulic conductivity; however, ‘Tucano’ plants plateaued at >80 % of PLC at ψ_stem_ close to −2 MPa, whereas ‘San Martino’ poplars plateaued at around −1.5 MPa.

**Fig. 4.**
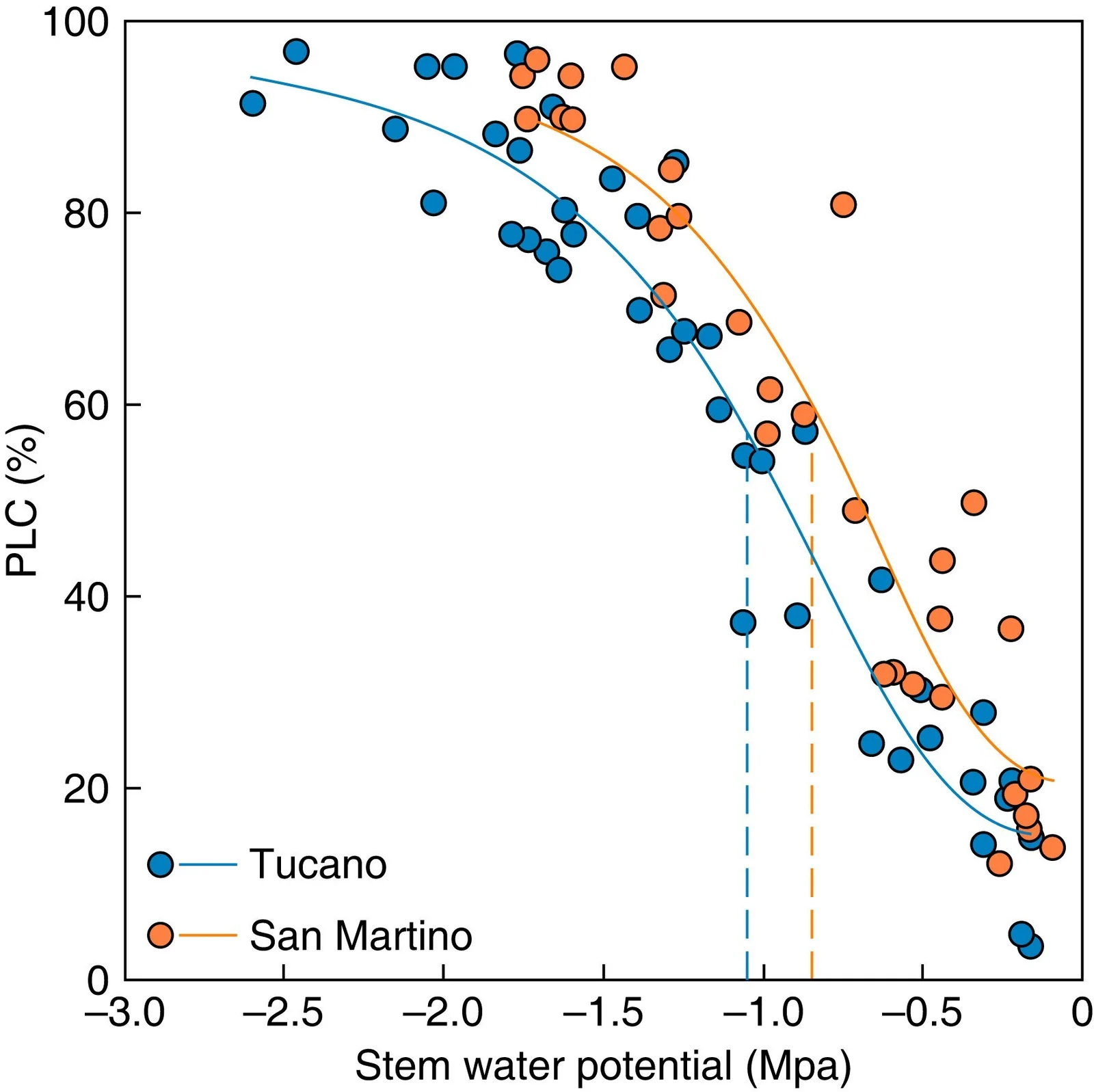
Percentage loss of hydraulic conductivity (PLC) in stems of ‘Tucano’ and ‘San Martino’ clones in relationship to stem water potential. Data were fitted with the four-parameter logistic curves (dose–response curve; blue line for ‘Tucano’ and orange line for ‘San Martino’) of the form: PLC = minPLC + (maxPLC − minPLC)/[1 + (Ψ/P50_PLC_ slope)], where minPLC is the minimum PLC in well-watered plants, maxPLC is set to 100 %, P50_PLC_ represents a 50 % loss of initial functionality [(minPLC + (maxPLC − minPLC); half maximal effective concentration; in our case, effective stem water potential], and slope describes the rate of PLC increase at P50_PLC_. Blue and orange dashed lines indicate P50 for the ‘Tucano’ and ‘San Martino’ clones, respectively. The statistical results of the *Z*-test are reported in Supplementary Data Table S1.

The 50 % loss of effective stem conductivity (P50) occurred at −1.05 MPa (s.e. = 0.0511, *t* = 20.626, *P* = 0.0000987) for ‘Tucano’ poplars and at −0.85 MPa (s.e. = 0.0673, *t* = 12.64, *P* < 0.0001) for ‘San Martino’ plants. The P50 parameter differed significantly between the two clones (*Z*-test; *t* = 2.41, d.f. = 73, *P* = 0.0053) (Paternoster *et al.*, 1998), indicating that stems of ‘San Martino’ clone were more vulnerable to embolism formation than those of the ‘Tucano’ clone.

The response of stomatal conductance (*g*_s_) to stem water potential also differed between the two clones (Supplementary Data Fig. S4). ‘San Martino’ plants closed their stomata more gradually as water stress increased, whereas ‘Tucano’ poplars exhibited a rapid stomatal closure at the onset of mild stress, around −0.6 MPa, well before stems reached the P50 value.

### Wood anatomical measurements

Xylogenesis remained active throughout the experiment, resulting in the formation of an incomplete tree ring ∼0.6–0.8 mm wide between planting and sampling (88 days), thereby providing a general indication of growth rates during the experimental period. Wood anatomical analyses revealed that, compared with ‘San Martino’, the ‘Tucano’ clone has intrinsically a greater specific theoretical hydraulic conductivity (*t* = −3.3936, d.f. = 4.9371, *P* = 0.01977), higher vessel density (∼14 % higher; *t* = −3.0655, d.f. = 5.4574, *P* = 0.02492) and a greater number of vessels per group (44 % higher; *t* = −5.9014, d.f. = 5.258, *P* = 0.001673), particularly in the two-vessel groups (*t* = −2.815, d.f. = 5.9839, *P* = 0.03064; Fig. 5).

**Fig. 5.**
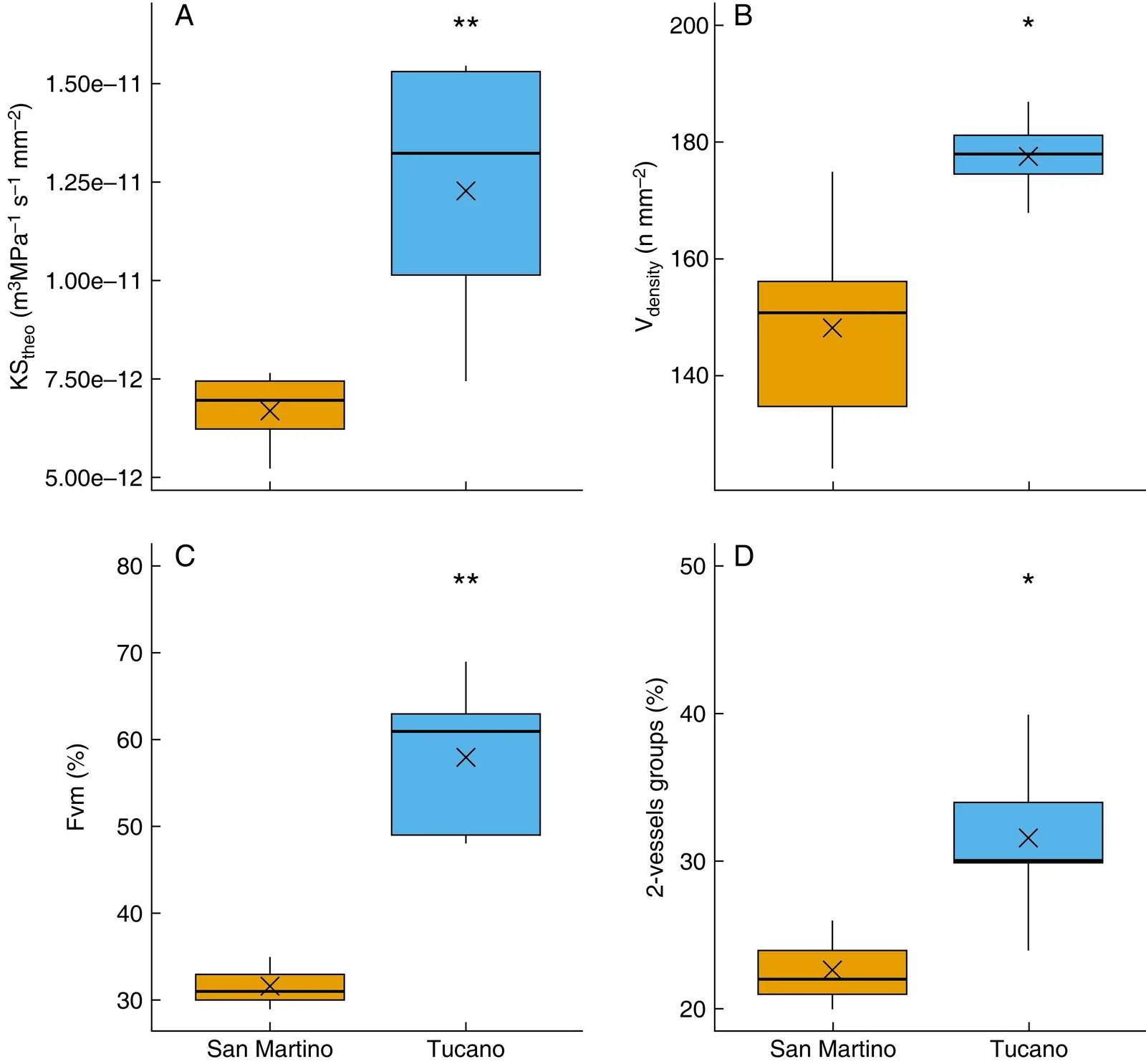
Wood anatomical parameters of stem xylem in ‘San Martino’ and ‘Tucano’ control plants related to hydraulic transport: (A) specific theoretical hydraulic conductivity (Ks_theo_); (B) vessel density (V_density_); (C) vessel multiple fraction (Fvm); and (D) 2-vessels group. The ‘×’ symbol represents the mean value. Asterisks indicate significance based on *t*-test analysis (*P < 0.05 and **P < 0.01). The statistical results are reported in Supplementary Data Table S1.

In addition, the ‘Tucano’ plants exhibited inherently higher wood density (∼6.5 % higher, *t* = −2.2927, d.f. = 21.106, *P* = 0.03224; Fig. 6A) and thicker xylem cell walls (∼15 % higher, *t* = −5.2479, d.f. = 6.4391, *P* = 0.001546; Fig. 6B). However, there were no significant differences between the clones in anatomical parameters related to vessel lumen size (mean vessel area, area-weighted average conduit diameter; Supplementary Data Fig. S5A, B) or in vessel grouping for the other common vessel groups (i.e. groups of three and four vessels; *P* > 0.05; Supplementary Data Fig. S5C, D). Finally, both clones were found inherently to possess a similar degree of packing, very close to the packing limit (Supplementary Data Fig. S6), thus achieving an efficient ratio between the total potential space dedicated to water transport and that dedicated to structural support.

**Fig. 6.**
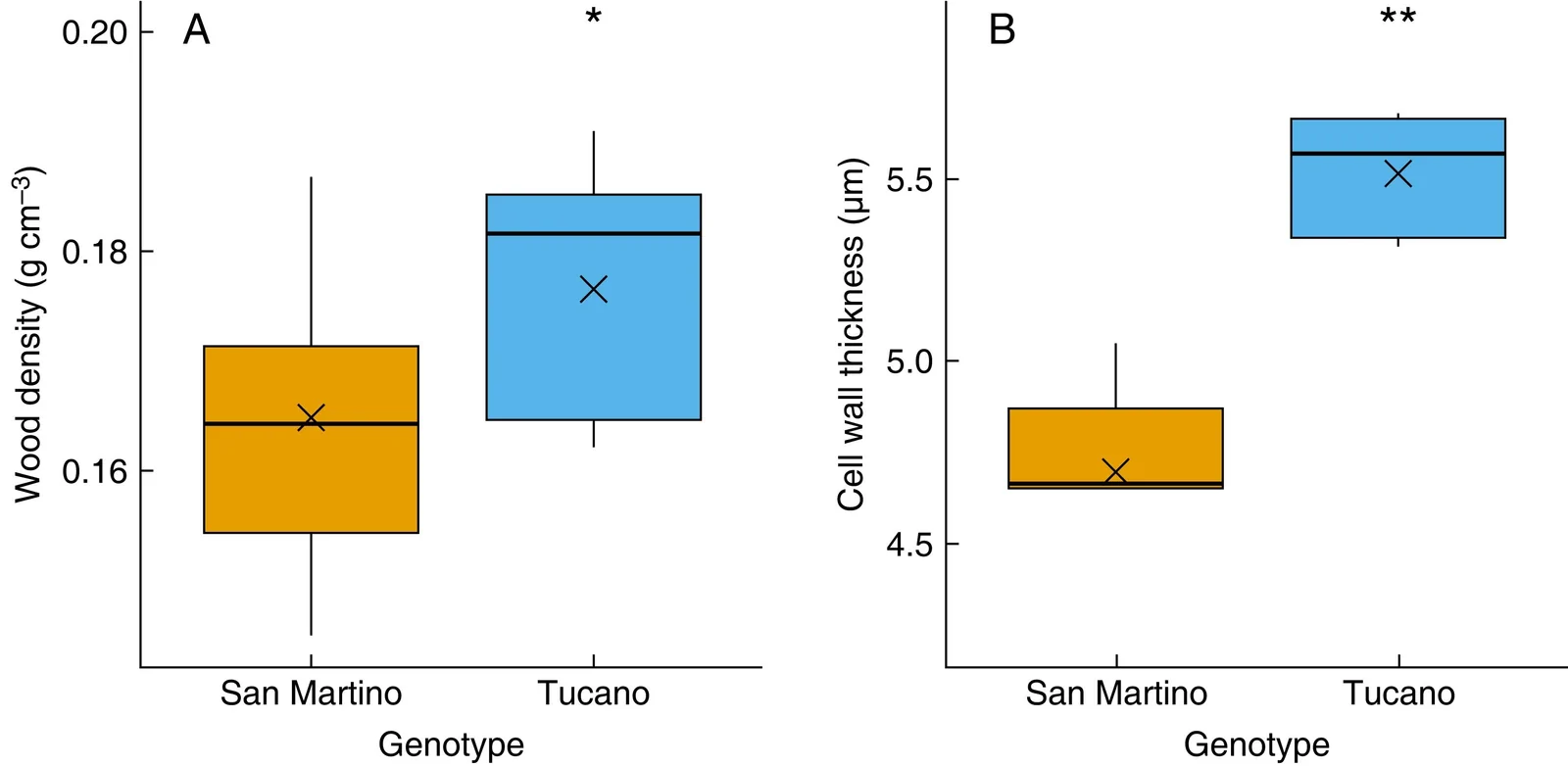
Wood anatomical parameters of stem xylem in ‘San Martino’ and ‘Tucano’ control plants: (A) wood density; and (B) xylem cell wall thickness. The ‘×’ symbol represents the mean value. Asterisks indicate significance based on *t*-test analysis (*P < 0.05 and **P < 0.01). The statistical results are reported in Supplementary Data Table S1.

### Soluble sugar and starch contents in stem

Prolonged drought slightly affected the accumulation of soluble sugars in the stems of the ‘Tucano’ clone, which exhibited a little higher NSC content, with sugars accumulation increasing as water stress intensified. A similar trend was observed in the stems of ‘San Martino’; however, the NSC concentration in this clone was in general lower than that measured in ‘Tucano’ stems (Fig. 7). Water stress also affected starch accumulation, but with the opposite trend: the treatment led to a slight reduction of starch content in ‘Tucano’ stems, with the lower starch concentration observed at the highest water stress level (lowest stem water potential). Interestingly, this trend was not observed in ‘San Martino’ samples, which did not show significant changes in NSC content (Fig. 7).

**Fig. 7.**
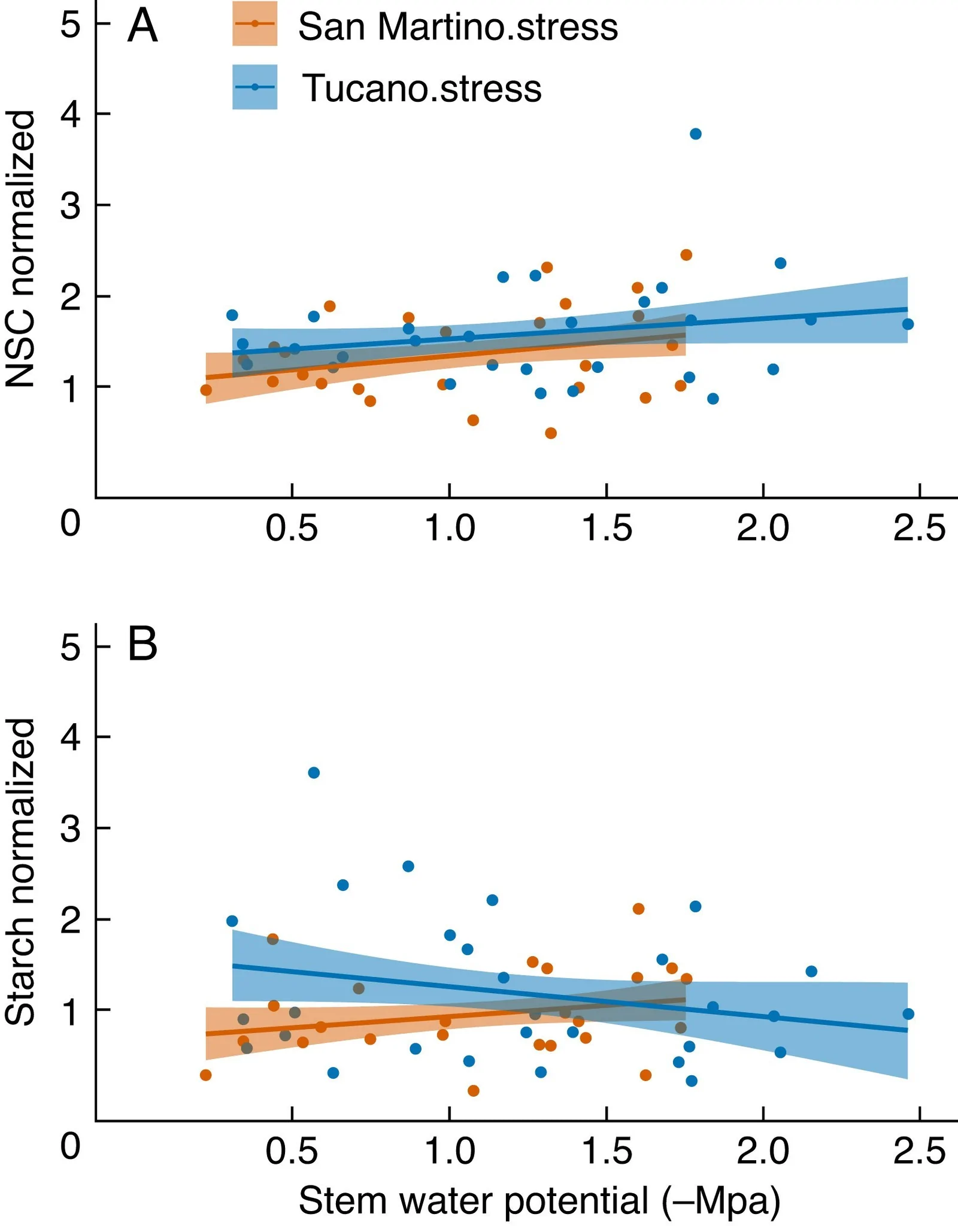
Measurements of non-structural carbohydrate (NSC; A) and starch content (in milligrams per gram; B) in the stems of stressed ‘Tucano’ and ‘San Martino’ poplars, normalized to their respective controls along the water stress gradient. Single points represent biological replicates, lines indicate linear regression. A linear regression model (*y* = *ax* + *b*) was fitted, and the coefficient of determination (*r*^2^) was used to evaluate the fit of the model. NSC linear regression for ‘San Martino’: intercept = 0.99, slope = 0.31, *r*^2^ = 0.09; NSC linear regression for ‘Tucano’: intercept = 1.32, slope = 0.18, *r*^2^ = 0.03; starch linear regression for ‘San Martino’: intercept = 0.66, slope = 0.24, *r*^2^ = 0.05; starch linear regression for ‘Tucano’: intercept = 1.53, slope = −0.29, *r*^2^ = 0.04.

## DISCUSSION

The duration, intensity and rate of progression of drought events influence growth, physiological performance and recovery capacity of trees differently (Trifilò *et al.*, 2017; Morabito *et al.*, 2025). We investigated the responses of two poplar clones to drought under gradually developing water limitation, creating a scenario that accurately reflects real-field situations in which larger soil volumes provide a higher water buffer, delaying the onset of stress. This experimental design allowed us to monitor the physiological dynamics and biochemical adjustments associated with drought acclimatization, rather than acute stress responses typically induced by rapidly imposed stress (Morabito *et al.*, 2022). Such an approach is particularly relevant for poplars, which are highly sensitive to water deficit yet exhibit marked genotypic variability in their drought responses (Fichot *et al.*, 2015).

Both ‘San Martino’ and ‘Tucano’ clones progressively reached severe water stress, with stem water potential dropping by about −1.3/−1.8 MPa over 3 weeks, consistent with previous observations in poplars exposed to long-term drought development (Tricerri *et al.*, 2025). ‘Tucano’ plants exhibited higher water consumption than ‘San Martino’ plants, attributable to a higher growth rate and greater transpiration. In ‘Tucano’, most water loss occurred during the first 12 days of stress development, coinciding with sustained growth during limiting water availability, whereas growth became more limited at later stages. In contrast, the growth of ‘San Martino’ plants was more affected by the treatment, exhibiting constrained growth from the outset.

In well-watered conditions, both clones exhibited similar levels of embolism formation; however, the rate at which stress was imposed significantly affected the amount of non-functional vessels. ‘Tucano’ was less vulnerable to xylem embolism than ‘San Martino’, reaching a 50 % loss of PLC at a more negative stem water potential (−1.05 vs. −0.85 MPa for ‘Tucano’ and ‘San Martino’, respectively). Nevertheless, both clones fell within the vulnerable range characteristic of the genus *Populus*, whose species are among the most cavitation-prone woody plants (mean P50 ≈ −1.44 MPa; Fichot *et al.*, 2015). The P50 values reported here are consistent with the broad inter- and intraspecific variability observed in poplars species and hybrid crosses, spanning from resistant species, such as *Populus tremuloides* (P50 = −2.13 MPa; Sperry and Sullivan, 1992), to highly vulnerable ones, such as *Populus euphratica* (P50 = −0.70 MPa; Hukin *et al.*, 2005), thus supporting the potential for improving drought resistance through genotype selection.

Differences in embolism resistance were closely linked to contrasting stomatal regulation strategies and intrinsic xylem anatomical structure. ‘Tucano’ exhibited a rapid and pronounced reduction in stomatal conductance as soil water availability declined, consistent with an avoidance strategy aimed at limiting xylem tension and preventing embolism formation and providing a larger hydraulic safety margin (Marron *et al.*, 2003; Regier *et al.*, 2009; Garavillon-Tournayre *et al.*, 2018). Such stomatal control is commonly reported to be one of the earliest responses to water limitation in poplars and other woody species (Tardieu and Simonneau, 1998; Bogeat-Triboulot *et al.*, 2007). This behaviour is likely to explain why, despite a faster decline in soil water content in ‘Tucano’ pots, driven by a larger transpiring surface, stem water potential remained comparable to that of ‘San Martino’ for a substantial duration of the experiment (12 days). Early stomatal closure therefore allowed ‘Tucano’ to maintain hydraulic functionality and delay the onset of critical water potentials, highlighting the importance of stomatal sensitivity as a key component of drought avoidance in poplars (Silim *et al.*, 2009).

In contrast, ‘San Martino’ maintained higher stomatal conductance during the first 12 days of the treatment, promoting the photosynthetic activity without an accompanying decline in stem water potential. However, this potential carbon gain was not translated into increased growth.

As expected, the rapid stomatal response in ‘Tucano’ was accompanied by a marked reduction in net CO_2_ assimilation. Nevertheless, ‘Tucano’ maintained relatively high iWUE during the early and intermediate phases of prolonged stress, indicating an effective balance between assimilation and stomatal limitation. Similar increases in iWUE under drought have been widely reported in poplar genotypes and are considered an acclimatization mechanism for survival under decreasing water availability (Monclus *et al.*, 2006; Cao *et al.*, 2014; Abdureyim *et al.*, 2025). Given that iWUE reflects the ratio between dry biomass produced and water used in transpiration (Gentilesca *et al.*, 2021), this trend in iWUE is reflected in the higher diameter growth rate observed in ‘Tucano’ plants subjected to water stress compared with those of the ‘San Martino’ poplars, supporting the idea that drought tolerance does not necessarily imply reduced productivity, but rather a re-optimization of carbon and water economics (Fichot *et al.*, 2015).

Responses of NSC pools to drought are notoriously variable in trees, ranging from accumulation to depletion or no change at all (Hartmann and Trumbore, 2016). The presence of xylem embolism can alter the carbohydrate metabolism and the partitioning between starch and soluble sugars in stem tissues (Salleo *et al.*, 2009; Secchi and Zwieniecki, 2011; Tomasella *et al.*, 2017), sometimes resulting in the accumulation of high NSC content in trees subjected to short-term drought events (Trifilò *et al.*, 2017). In *Populus tremula* × *alba*, it has been observed that rapid and severe stress promotes starch degradation in stems and increases the concentrations of soluble carbohydrates, thereby providing the osmotic potential necessary for the recovery of embolized vessels (Pagliarani *et al.*, 2019).

In the present study, ‘Tucano’ exhibited slightly higher overall NSC concentrations than ‘San Martino’. Although ‘Tucano’ showed a trend towards increasing soluble sugar content with declining stem water potential, neither clone exhibited strong regulation of carbohydrate reserves across drought levels. Under severe stress, the ‘Tucano’ clone displayed lower starch contents in stem tissues, suggesting that carbohydrates were either used to sustain vegetative growth rather than stored as reserves or were rapidly degraded to increase the soluble NSC pool. Overall, NSC and starch contents in stems showed low explanatory power for stem water potential. This contrasts with studies reporting sugar accumulation as a key mechanism for osmotic adjustment (Tschaplinski and Tuskan, 1994; Marron *et al.*, 2002) and instead supports observations that some poplar clones rely on avoidance strategies, such as stomatal regulation, leaf abscission and growth reduction, rather than osmotic regulation (Gebre *et al.*, 1998). The ‘San Martino’ clone resorted to leaf shedding as a last-ditch strategy to avoid hydraulic failure; indeed, it began to shed its leaves approximately at −1.5 MPa. It is also possible that osmotic adjustment in these clones relies less on sugar and more on other osmolytes, such as proline, other amino acids and/or polyols (Haghpanah *et al.*, 2024).

The observed hydraulic and physiological differences are consistent with the anatomical traits of the two clones. Our study was not designed to quantify the anatomical features in response to drought stress, hence, to isolate anatomical variation attributable to intrinsic genetic differences, analyses were conducted on control plants. Furthermore, all stem segments were sampled at a fixed distance from the main apical bud, thereby minimizing the influence of axial variation in conduit dimensions associated with plant size and height (Olson *et al.*, 2014, 2021). The ‘Tucano’ clone showed several wood anatomical traits that are intrinsically different from those of ‘San Martino’ and are likely to have contributed to its physiological response to water stress. Specifically, ‘Tucano’ exhibited higher specific theoretical hydraulic conductivity, greater vessel density and increased vessel clustering, resulting in a xylem architecture composed of numerous highly interconnected vessels. Such an anatomical configuration has been shown to influence hydraulic function and connectivity in woody plants, with aggregated networks predicted to enhance both connectivity and conductivity (Martínez-Vilalta *et al.*, 2012). Furthermore, such a network can sustain hydraulic redundancy within the xylem, facilitating lateral water redistribution through pit pairs (Rodriguez-Zaccaro *et al.*, 2021). Increased vessel frequency and reduced vessel diameter are common acclimatization responses to drought in poplars and other woody species, allowing the maintenance of water transport while limiting embolism risk (Sperry *et al.*, 2006; Yu *et al.*, 2021).

The ‘Tucano’ clone was also found to be predisposed genetically to produce denser wood, primarily owing to thicker cell walls, reflecting a greater carbon investment in stem structure. Higher wood density and thicker cell walls are frequently associated with enhanced embolism resistance and greater mechanical strength (Hacke and Sperry, 2001; Jacobsen *et al.*, 2007), albeit at a potential carbon cost. This trade-off between hydraulic safety and carbon investment is well documented in poplars and other fast-growing species (Cochard *et al.*, 2007; Wang and Wang, 2025). From an applied perspective, higher wood density is also a desirable trait for cellulose production (Ona *et al.*, 1998; Martins *et al.*, 2020; Nazari *et al.*, 2020), further reinforcing the agronomic value of ‘Tucano’.

Overall, the coordinated adjustments in stomatal behaviour, hydraulic architecture, carbon allocation and growth indicate that the ‘Tucano’ clone adopts avoidance strategies in response to prolonged water deficit (Marron *et al.*, 2003; Garavillon-Tournayre *et al.*, 2018) and is better acclimatized to water stress than ‘San Martino’. Although avoidance strategies are often associated with reduced productivity, our results show that in gradual and prolonged drought conditions, ‘Tucano’ successfully reconciles hydraulic safety with growth and wood quality. In contrast, ‘San Martino’ appears more vulnerable to hydraulic failure and less efficient at maintaining growth under water limitation.

In the context of future climate scenarios characterized by more frequent and prolonged drought events, these findings support the selection of poplars that combine tolerance to both biotic and abiotic stresses. From both ecological and silvicultural perspectives, the ‘Tucano’ clone emerges as a more suitable candidate than ‘San Martino’ for poplar cultivation under increasingly water-limited conditions, offering improved drought resilience without compromising productivity or wood quality.

## Supplementary Material

mcag254_Supplementary_Data

## SUPPLEMENTARY DATA

Supplementary data are available at *Annals of Botany* online and consist of the following. Figure S1: daily greenhouse meteorological data of the period before and during the drought experiment. Figure S2: region of interest selected for anatomical analysis of a stem cross-section from a control plant and corresponding analysis performed using CARROT software. Figure S3: percentage of temporal stem diameter growth of ‘Tucano’ and ‘San Martino’ clones in control and drought conditions. Figure S4: relationship between stomatal conductance and stem water potential during prolonged water stress for the ‘Tucano’ and ‘San Martino’ clones. Figure S5: additional wood anatomical traits measured in control plants of ‘Tucano’ and ‘San Martino’ clones. Figure S6: vessel density vs. area-weighted mean conduit diameter in control plants of ‘Tucano’ and ‘San Martino’ clones. Table S1: statistical report. Table S2: biometric traits measured in well-watered poplar clones.
