# Supplementary material for: Two clones, two paths: divergent intrinsic xylem traits and hydraulic responses to prolonged drought in poplar
Source: Ann Bot. 2026 Aug 12;138(5):2057–69. doi: 10.1093/aob/mcag254 (PMC13715041; doi:10.1093/aob/mcag254)
Supplement: Supplementary file 1 [file mcag254_supplementary_data.docx]

Type of article: Research Article

**Title: Two Clones, Two Paths: divergent intrinsic xylem traits and hydraulic responses to prolonged drought in poplar**

**Supplementary Material**

**Figure S1.** Daily greenhouse meteorological data (A-B) and daily Photosynthetically Active Radiation (C) of the growing period before the experiment and during the prolonged drought stress highlighted in yellow. Red, black and blue points denote daily maximum, mean and minimum values respectively.

**Figure S2.** Percentage of temporal stem diameter growth of ‘Tucano’ and ‘San Martino’ poplar clones under control and drought conditions. Points represent mean values and bars denote s.d. One-way ANOVA tests significant differences between plant groups for sampling time; different letters denote nonhomogeneous groups (P < 0.05) based on the Tuckey post hoc test. Results of ANOVA for each sampling day from the beginning of the experiment: 0 day: F=NaN, degree of freedom [df]=3, P=NaN; 13 days: F= 4.769e+31, degree of freedom [df]=3, P= <2e-16; 20 days: F= 3.429e+31, degree of freedom [df]=3, P=<2e-16; 27 days: F=3.511e+31, degree of freedom [df]=3, P=<2e-16.

**Table S1.** Statistical results. df= degree of freedom; Significant codes: ‘***’ P<0.001; ‘**’ P<0.01; ‘*’ P<0.05; ‘ns’ P>0.05. DI indicates drought imposition.

| **Graph** | **Statistical test** | **Bin** | **t** | **F** | **df** | **P** | **Significance code** |
| --- | --- | --- | --- | --- | --- | --- | --- |
| 1 | One-way ANOVA | DI: 0-6 days | - | 1.378 | 3 | 0.268 | ns |
| 1 | One-way ANOVA | DI:6-12 days | - | 5.029 | 3 | 0.00412 | ** |
| 1 | One-way ANOVA | DI:12-18 days | - | 22.02 | 3 | 9.86e-08 | *** |
| 1 | One-way ANOVA | DI:18-22 days | - | 108.2 | 3 | 1.9e-14 | *** |
| 2A | Kruskal-Wallis | 1 day | - | 52.710 | 3 | 2.11e-11 | *** |
| 2A | Kruskal-Wallis | 2 day | - | 53.427 | 3 | 1.48e-11 | *** |
| 2A | Kruskal-Wallis | 4 day | - | 54.024 | 3 | 1.10e-11 | *** |
| 2A | Kruskal-Wallis | 5 day | - | 38.996 | 3 | 1.73e-08 | *** |
| 2A | Kruskal-Wallis | 6 day | - | 54.217 | 3 | 1.00e-11 | *** |
| 2A | Kruskal-Wallis | 7 day | - | 54.082 | 3 | 1.07e-11 | *** |
| 2A | Kruskal-Wallis | 8 day | - | 52.876 | 3 | 1.94e-11 | *** |
| 2A | Kruskal-Wallis | 9 day | - | 50.307 | 3 | 6.87e-11 | *** |
| 2A | Kruskal-Wallis | 10 day | - | 50.308 | 3 | 6.86e-11 | *** |
| 2A | Kruskal-Wallis | 11 day | - | 38.222 | 3 | 2.53e-08 | *** |
| 2A | Kruskal-Wallis | 12 day | - | 41.243 | 3 | 5.80e-09 | *** |
| 2A | Kruskal-Wallis | 13 day | - | 35.196 | 3 | 1.10e-07 | *** |
| 2A | Kruskal-Wallis | 14 day | - | 35.939 | 3 | 7.71e-08 | *** |
| 2A | Kruskal-Wallis | 15 day | - | 32.459 | 3 | 4.18e-07 | *** |
| 2A | Kruskal-Wallis | 16 day | - | 28.341 | 3 | 3.07e-06 | *** |
| 2A | Kruskal-Wallis | 17 day | - | 25.862 | 3 | 1.01e-05 | *** |
| 2A | Kruskal-Wallis | 18 day | - | 24.691 | 3 | 1.79e-05 | *** |
| 2A | Kruskal-Wallis | 19 day | - | 20.513 | 3 | 0.00013 | *** |
| 2A | Kruskal-Wallis | 20 day | - | 15.506 | 3 | 0.00143 | *** |
| 2B | *t*-test | 1 day | -4.698 | - | 73. 3512 | 1.19e-05 | *** |
| 2B | *t*-test | 2 day | -4.952 | - | 74.3674 | 4.46e-06 | *** |
| 2B | *t*-test | 4 day | -4.680 | - | 74.999 | 1.24e-05 | *** |
| 2B | *t*-test | 5 day | -4.525 | - | 74.9202 | 2.22e-05 | *** |
| 2B | *t*-test | 6 day | -3.242 | - | 58.3291 | 0.0019 | ** |
| 2B | *t*-test | 7 day | -1.771 | - | 64.3938 | 0.081 | ns |
| 2B | *t*-test | 8 day | -3.440 | - | 62.3074 | 0.001 | ** |
| 2B | *t*-test | 9 day | -0.008 | - | 66.9578 | 0.993 | ns |
| 2B | *t*-test | 10 day | -1.889 | - | 59.2579 | 0.063 | ns |
| 2B | *t*-test | 11 day | 8.389 | - | 63.5933 | 6.96e-12 | *** |
| 2B | *t*-test | 12 day | 0.920 | - | 59.8286 | 0.3612 | ns |
| 2B | *t*-test | 13 day | 5.690 | - | 51.9771 | 5.90e-07 | *** |
| 2B | *t*-test | 15 day | 0.830 | - | 30.6570 | 0.4128 | ns |
| 2B | *t*-test | 16 day | 0.694 | - | 31.9819 | 0.4922 | ns |
| 2B | *t*-test | 17 day | 2.331 | - | 30.6919 | 0.0264 | * |
| 2B | *t*-test | 18 day | 0.796 | - | 21.4571 | 0.4345 | ns |
| 2B | *t*-test | 19 day | 1.544 | - | 20.8936 | 0.1374 | ns |
| 2B | *t*-test | 20 day | 0.191 | - | 10.1092 | 0.8517 | ns |
| 2B | *t*-test | 21 day | 2.5 | - | 5.0894 | 0.0536 | ns |
| 3A | One-way ANOVA | DI: 0-6 days | - | 1.897 | 3 | 0.149 | ns |
| 3A | One-way ANOVA | DI: 6-12 days | - | 11.37 | 3 | 7.22e-06 | *** |
| 3A | One-way ANOVA | DI: 12-18 days | - | 13.22 | 3 | 1.96e-05 | *** |
| 3A | One-way ANOVA | DI: 18-22 days | - | 41.67 | 3 | 2.87e-10 | *** |
| 3B | One-way ANOVA | DI:0 -6 days | - | 3.851 | 3 | 0.0178 | * |
| 3B | One-way ANOVA | DI: 6-12 days | - | 13.59 | 3 | 1.08e-06 | *** |
| 3B | One-way ANOVA | DI: 12-18 days | - | 31.72 | 3 | 7.67e-09 | *** |
| 3B | One-way ANOVA | DI: 18-22 days | - | 133.8 | 3 | 2.56e-16 | *** |
| 3C | One-way ANOVA | DI: 0-6 days | - | 0.717 | 3 | 0.549 | ns |
| 3C | One-way ANOVA | DI: 6-12 days | - | 8.501 | 3 | 0.000105 | *** |
| 3C | One-way ANOVA | DI: 12-18 days | - | 3.935 | 3 | 0.0193 | * |
| 3C | One-way ANOVA | DI: 18-22 days | - | 1.209 | 3 | 0.326 | ns |
| 4 | Z-test | - | 2.41 | - | 73 | 0.0053 | ** |
| 5A | *t*-test | - | -3.3936 | - | 4.9371 | 0.01977 | ** |
| 5B | *t*-test | - | -3.0655 | - | 5.4574 | 0.02492 | * |
| 5C | *t*-test | - | -5.9014 | - | 5.258 | 0.001673 | ** |
| 5D | *t*-test | - | -2.815 | - | 5.9839 | 0.03064 | * |
| 6A | *t*-test | - | -2.2927 | - | 21.106 | 0.03224 | * |
| 6B | *t*-test | - | -5.2479 | - | 6.4391 | 0.001546 | ** |

**Table S2.** Biometric traits measured in well-watered poplar clones. Plant height and total leaf number were recorded at the end of the experiment (n=5 per clone). Mean leaf area was determined from individual leaves sampled across plants (n=23 per clone).

|  | **TUCANO** | **SAN MARTINO** | **P** |
| --- | --- | --- | --- |
| Final height (cm) | 105.70 ± 10.52 a | 91.80 ± 4.2 b | 0.0253 |
| Number of leaves | 29.2 ± 5.93 a | 16.2 ± 2.3 b | 0.0018 |
| Mean leaf area (cm^2^) | 112.46 ± 25.13 a | 127.49 ± 26.74 a | 0.0615 |

Values are means ± s.d. Different lower case letters following s.d. values indicate significant differences attested by *t*-test (P < 0.05).

**Figure S3.** (A) Region of interest selected for anatomical analysis of a stem cross-section from a control plant and (B) corresponding analysis performed using CARROT software. Magnification: 100x.

**Figure S4.** Stomatal conductance (gs) in relation to stem water potential during prolonged stress for the ‘Tucano’ (blue dots) and ‘San Martino’ (orange dots) clones. Each point represents a biological replicate. Data were fitted with four-parameter logistic curves using the same function described for the vulnerability curves (Fig. 4).

**Figure S5.** Stem xylem anatomical traits of control plants of two poplar clones (‘Tucano’ and ‘San Martino’): (A) mean vessel lumen area (LA). (B) area-weighted mean vessel diameter (dA). (C) percentage of vessels in 3-vessels groups (3-vessels groups). (D) percentage of vessels in 4-vessels groups (4-vessels groups). The x sign represents the mean value. No significant difference was found in these parameters between the two clones. Results of *t*-test: LA: t=0.075802, degree of freedom [df]= 6.7768, P=0.9418; dA: t= 0.13127, degree of freedom [df]= 6.573, P= 0.8995; 3-vessels groups: t= -1.6784, degree of freedom [df]= 5.7073, P=0.1468; 4-vessels groups: t=-2.3591, degree of freedom [df]=4, P=0.07774.

**Figure S6.** Vessel density (V_density_) vs. area-weighted mean conduit diameter (dA) in control plants of two poplar clones (‘Tucano’ and ‘San Martino’). Each data point represents one plant. The heavy solid line shows the geometric packing limit of the maximum number of conduits of a given diameter per mm^2^. assuming square packing. The square-packing limit was calculated as 1 ⁄ *d^2^*. The axes are shown on log-log plots. The small graph at the bottom left shows a zoom of the points.
